# Frequency-specific microcurrent improves hand function and Raynaud’s symptoms in scleroderma: results of two pilot studies

**DOI:** 10.1093/rheumatology/keaf301

**Published:** 2025-06-04

**Authors:** Walter M Gregory, Katherine Bagley, Sookhoe Eng, Carolyn McMakin, Francesco Del Galdo

**Affiliations:** Division of Clinical Medicine, University of Sheffield, Sheffield, UK; Chapel Allerton Osteopath, Leeds, UK; University of Leeds Faculty of Medicine and Health, Leeds Institute of Rheumatic and Musculoskeletal Medicine, Leeds, UK; Fibromyalgia and Myofascial Pain Clinic of Portland, Portland, OR, USA; University of Leeds Faculty of Medicine and Health, Leeds Institute of Rheumatic and Musculoskeletal Medicine, Leeds, UK

**Keywords:** microcurrent, FSM, scleroderma, sclerosis, Raynaud’s, Cochin, scarring, frequencies, capillaries, skin

## Abstract

**Objectives:**

To evaluate the efficacy of frequency-specific microcurrent as a treatment designed to improve hand function and alleviate Raynaud’s symptoms in patients with systemic sclerosis.

**Methods:**

Seventeen patients were treated in two separate pilot studies for 60 and 45 min, respectively, over 1 or 2 days with microcurrent frequencies specific to scarring in the hands and capillaries, and to Raynaud’s disease. Efficacy was evaluated using the Cochin hand function score questionnaire before and after treatment, and a visual analogue scale to evaluate the severity of the Raynaud’s symptoms.

**Results:**

Improvement in hand function was observed in both studies, being significant in the second pilot study with an average improvement of 38% (95% CI 22%–55%, *P* = 0.002) and in both studies combined with an average improvement of 40% (95% CI 26%–55%, *P = *0.0001). There was a significant improvement in mean Raynaud’s VAS scores pre- and post-treatment of 18 points (out of 100), 95% CI 3.3–33 points, *P = *0.016. A proportion of patients achieved very substantial improvements in hand function, with particular tasks improving from being nearly impossible to do to being able to be performed without difficulty.

**Conclusions:**

The degree of improvement in hand function obtained over a very short treatment period of 45 min to 1 h was significant. Further randomized, controlled studies are needed to confirm these results, and longer-term follow-up will be required to establish whether the changes are maintained and whether repeated treatments can produce further improvements.

Rheumatology key messages

- Substantial and sustained improvements in hand function were observed after only an hour of treatment.
- The treatment is non-invasive, easy to deliver and can be repeated without side effects.
- Significant improvements were also seen in Raynaud’s symptoms over a short 40-min treatment period.

## Introduction

More than half of the patients with systemic sclerosis (scleroderma) are disabled from poor hand function for which treatment options are limited [1]. Frequency-specific microcurrent (FSM) treatment has previously been used effectively to treat scarring [2–4], so it was hypothesized that this might be an effective treatment for scleroderma. Microcurrent treatment uses physiologic subsensory current that has been shown to increase ATP [3–5]. Wet contacts connected to the microcurrent device conduct the current and frequencies so they flow through the affected area. FSM uses frequencies from a list found on a medical device made in 1922, which included a frequency to change ‘scar tissue’. For patients with scleroderma, scarring is the primary issue. Frequencies for scarring have been in use clinically since 1995 and several published papers show their efficacy in reducing nerve adhesions, dry macular degeneration and improving pediatric torticollis [6–8]. Scleroderma includes scarring in the capillaries and connective tissue [9, 10] for which frequencies were available from the 1922 list that were used in treating this patient group. No information is available on how the frequencies from the 1922 list were derived.

Almost all patients with systemic sclerosis also have Raynaud’s syndrome [11]. The rationale for also treating their Raynaud’s with FSM is given in Supplementary Data S1, available at *Rheumatology* online.

### Patients and methods

Scleroderma patients with reduced hand function, defined as having a positive Cochin hand function test score, were treated in two pilot studies with a CE-marked two-channel microcurrent medical device manufactured by Microcurrent Technologies (Seattle, WA, USA) delivering three-digit specific frequencies simultaneously on each of two channels at 100–200 microamperes current. All patients were members of a local scleroderma support group and consented to the treatment. In study 1, treatment was applied using microcurrent frequencies from the 1922 list [12] (see Supplementary Table S1, available at *Rheumatology* online) targeted at treating scarring of skin, connective tissue, capillaries, tendon sheaths and joint capsules. Frequency effectiveness was evaluated by the tissue softening produced [13]. When the frequency no longer softened the tissue, the frequency was changed. In study 1, a range of settings were used to establish which settings were the most useful. Frequency combinations that softened the tissue and continued to soften the tissue for minutes at a time were considered to be effective combination settings.

In study 1, patients were treated for approximately one hour on two consecutive days. Following encouraging results from this study, the second study was designed to use a simpler set of frequencies, focusing on those that had been most effective in study 1. The combination frequencies for scarring (13 Hz), sclerosis (3 Hz) and vitality (49 Hz) in the skin (355 Hz), connective tissue (77 Hz) and capillaries (162 Hz) produced the most change in the affected tissue. Following the first trial, several patients were treated in demonstrations to the local scleroderma group, and with additional one-on-one private treatments. These treatments established the rapid and effective response of the settings for scarring in the skin and capillaries. The frequency described as increasing ‘vitality’ in the skin and capillaries was used after the frequencies to relieve the known pathologies. In study 2, frequencies were used for about 40 min in total; 15 min for scarring in the skin and ∼5 min each for the other five frequency combinations. Details of the treatment are given in Supplementary Data S2, available at *Rheumatology* online.

In study 2, patients were also treated with a second microcurrent device using the frequencies 40 Hz and 562 Hz, thought to be effective for Raynaud’s (Supplementary Data S1, available at *Rheumatology* online). These frequencies were used simultaneously with the scarring frequencies for the duration of the 40-min treatment session. Follow-up was shorter in the second trial, as the results in the first trial showed the pattern of early change was sustained for a period of weeks.

Study 1 included six patients and was carried out between 19 September 2019 and 21 September 2019, at a complementary medicine venue in Chapel Allerton organized by the Leeds scleroderma support group. All patients were treated by C.M. Study 2 included 11 patients and was carried out on 17 January 2022, at the osteopathy/microcurrent clinic run by K.B. in Chapel Allerton. Patients were treated by either W.M.G. or K.B. Patients were identified from the HRA Strike study [14], which has ethical approval (Newcastle & North Tyneside 2 REC: 15.NE.0211), and as part of Strike, patients also consented to be approached for related research, including analysis of hand function using the Cochin questionnaire. Patients gave additional written informed consent for the microcurrent procedures.

All patients completed the Cochin hand function score questionnaire (CHFS) [15]. The magnitude of changes in the CHFS can be evaluated by reference to the minimally clinically important difference (MCID) in this score in scleroderma, which has been estimated by the Cochin group to be −3.38 [16]. For the evaluation of the Raynaud’s treatment a linear visual analogue scale (VAS) was used, as described in Merkel *et al.* [17]. Schedules and timings for administration of both scales (CHFS and VAS) are given in Supplementary Data S3 and S4, available at *Rheumatology* online.

### Statistical methods

Cochin hand function scores and VAS Raynaud’s scores were compared pre- and post-treatment, and at different times after treatment, using the exact version of the Wilcoxon matched pairs signed rank sum test [18]. Further statistical methods details are given in Supplementary Data S5, available at *Rheumatology* online.

## Results

### First pilot study

**Figure 1.**
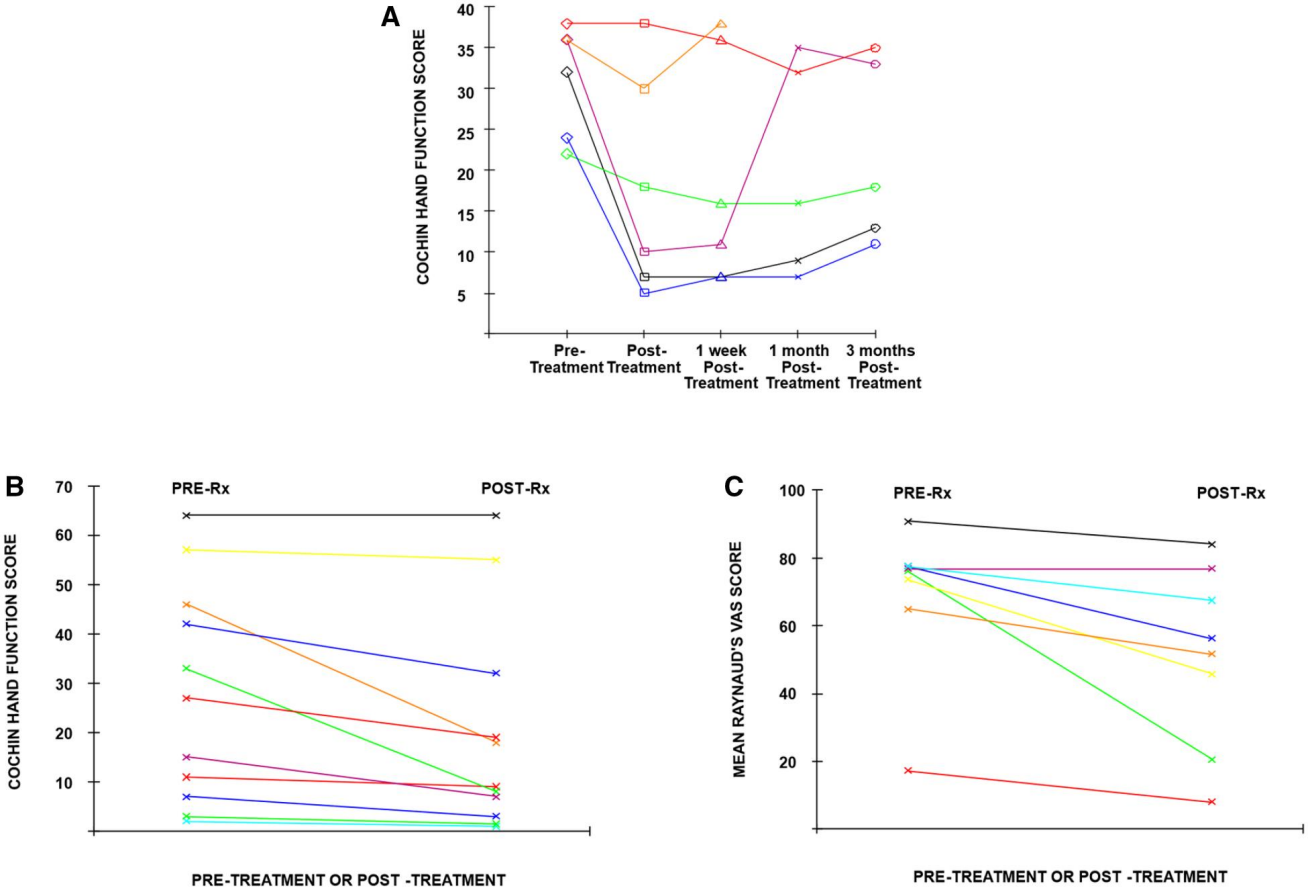
Changes in individual patient scores: (**A**) Cochin hand function scores for first pilot study; (**B**) Cochin hand function scores for second pilot study; (**C**) Raynaud’s VAS scores for second pilot study

An average improvement of 44% in hand function scores was seen during treatment. Three of the six patients exhibited very substantial improvements in hand function (see Fig. 1A) with improvements averaging 77% from the pre-treatment scores to the post-treatment scores a day later.

### Second pilot study

**Figure 2.**
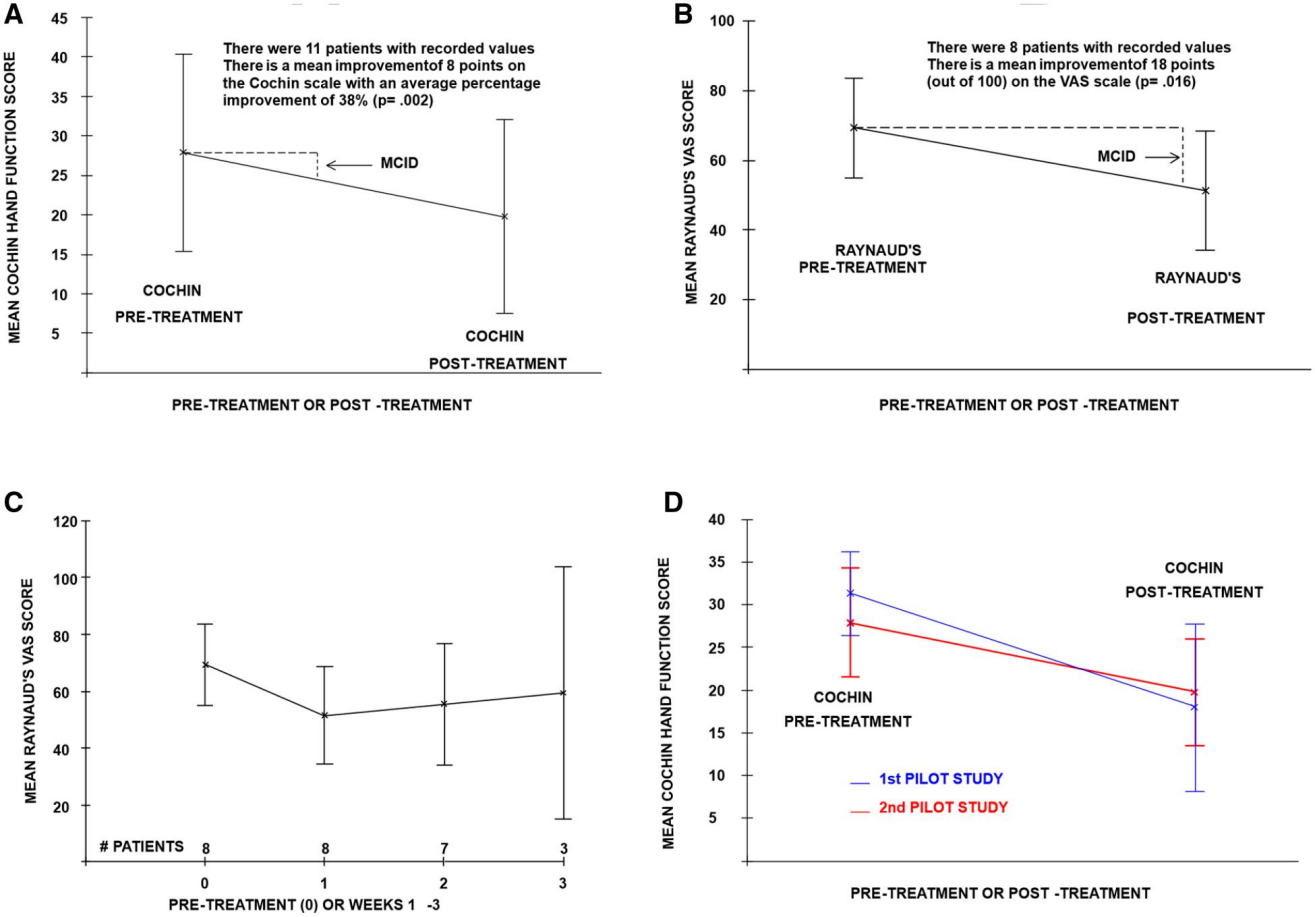
Mean changes in scores. (**A**) Cochin hand function scores for second pilot study; (**B**) Raynaud’s VAS scores for second pilot study; (**C**) Raynaud’s VAS scores over a three-week period for second pilot study; (**D**) Cochin hand function scores comparing first pilot study with second pilot study

The changes in individual hand function scores pre-and post-treatment are shown graphically in Fig. 1B, with mean pre-and post-treatment levels (±95% CIs) displayed in Fig. 2A. There was an average improvement in total Cochin hand function score of 8 points, corresponding to an improvement of 38% in the mean (95% CI 22%–55%, *P = *0.002, Wilcoxon test). Note that the improvements happened very quickly, over the duration of the 40-min treatment. Ten of the 11 patients showed improvement.

The changes in individual Raynaud’s VAS score are shown graphically in Fig. 1C with the mean VAS scores comparing pre-treatment with one week shown in Fig. 2B, which shows a mean improvement of 18 points out of 100 (95% CI 3.3–33 points, *P = *0.016, Wilcoxon test). Patient characteristics for the two studies are given in Supplementary Table S2, available at *Rheumatology* online.

### Pilot studies combined

Although the duration of treatment was longer (over 2 days rather than one day) in study 1 compared with study 2, changes appear to occur very quickly, so it was deemed reasonable to combine the results of the two pilot studies looking at mean changes pre-and post-treatment in the Cochin hand function score (Fig. 2D). Improvements were similar over the two studies, with overlapping confidence intervals on the means. With longer treatment, there was a slightly greater improvement in study 1, though the numbers are too small for this to be definitive. The combined pre-and post-treatment changes in the two studies showed a highly significant improvement of 40% in the mean Cochin hand function score (95% CI 26%–55%, *P = *0.0001, Wilcoxon test).

Responses to component questions of the Cochin hand function questionnaire are given in Supplementary Data S6D, available at *Rheumatology* online, and showed some remarkable changes, with two patients reporting improvements in the ability to pick up coins from a tabletop from ‘Nearly impossible to do’ before treatment to ‘Yes, without difficulty’ after treatment.

Further, more detailed results for both pilot studies and for the two pilot studies combined are given in Supplementary Data S6, available at *Rheumatology* online.

## Discussion

The degree of improvement in hand function after such a short treatment time was a surprise. That patients should have sufficiently large improvements after 40 min of treatment that they could now pick up coins from a tabletop easily, whereas previously this had been nearly impossible for them (see above and Supplementary Data S6, available at *Rheumatology* online), is very encouraging for this new form of treatment. Some patients repeated these tasks a number of times, expressing amazement that they could now perform them. The data in Supplementary Table S3, available at *Rheumatology* online suggests that after the microcurrent treatment patients had more feeling in their hands and fingers, and more dexterity, and so activities like buttoning a shirt or turning a round doorknob became easier. One patient later reported that she loved to sew, and had not been able to for some time, but the microcurrent treatment gave her the sensitivity and strength in her fingers to be able to sew again. Another patient, who was a musician, later reported that he was able to play the double bass again because his fingers now had the sensitivity to feel and manipulate the strings and their vibrations effectively. Activities that required more force or strength to be exerted, like cutting meat with a knife, or holding a bowl or a plateful of food, or grasping a full bottle and raising it were perhaps not affected to such a degree by the treatment. Treating with frequencies thought to address the capillaries may have improved blood perfusion to the sensory nerves at the fingertips; no frequencies thought to affect scarring in the nerves were used.

The lack of response in the two patients with very high Cochin hand function scores suggests that microcurrent treatment may be ineffective if the disease is too severe. The damage to the joints in the fingers may be too great for the treatment to affect them significantly. Otherwise, the treatment appears to show benefit even with patients having moderate hand function impairment.

The study was not randomized, and so it is possible that the responses were a placebo effect. Controlled trials are still needed to confirm that the treatment has genuine benefit. However, the magnitude and persistence of the improvements suggests that the treatment effect is real. The changes in hand function persisted out to 3 months in two patients in study 1 where we had this duration of follow-up. A placebo effect would likely be more short-lived and disappear once the patients were no longer in contact with the microcurrent practitioners and their enthusiasm for the treatment, though of course it cannot be ruled out.

These pilot trials had limited time and resources so there was no control group, no blinding and every patient was treated. It is possible that the observed improvements are attributable to placebo effects, expectation bias or confounders such as the attention paid to the patients from the practitioners. To counterbalance these obvious limitations, changes in function were both large and sustained in many of the patients who responded. Massage alone had been used in these patients previously with no sustained benefit, so the treatment could be tested against massage alone in a future controlled trial.

Although pre-and post-treatment comparisons gave highly significant *P*-values, the studies were also very small, and the confidence intervals were wide, reflecting the variability in individual responses and making it problematic to generalize too strongly from the results. The follow-up was also relatively short and it is possible that the effects of the microcurrent would fade over time. If so, repeated microcurrent treatments may be necessary to maintain the large improvements observed in the responders.

This new treatment approach shows great promise in a clinical manifestation that is not currently targeted by any intervention trial despite its social and healthcare burden [1]. It is easy to deliver, non-invasive and had no reported adverse effects. Future controlled trials could include sham microcurrent treatment, massage alone, more treatment sessions, longer-term follow-up and should be limited to patients with mild to moderate disease. Exclusionary criteria for this trial should be determined by the initial Colchin hand function score. All evaluations in these studies were subjective, and therefore potentially prone to bias. We intend to include additional objective measures in future studies, such as grip strength, capillaroscopy and thermography for Raynaud’s.

The dramatic results of these pilot studies suggest that further study of FSM is important to determine whether its short-term or long-term use could maintain or surpass the improvements seen in these studies.

## Supplementary material

Supplementary material is available at *Rheumatology* online.

## Supplementary Material

keaf301_Supplementary_Data

## Data Availability

The data underlying this article will be shared on reasonable request to the corresponding author.
