# Supplementary material for: Frequency-specific microcurrent improves hand function and Raynaud’s symptoms in scleroderma: results of two pilot studies
Source: Rheumatology (Oxford). 2025 Jun 4;64(10):5504–8. doi: 10.1093/rheumatology/keaf301 (PMC12494225; doi:10.1093/rheumatology/keaf301)
Supplement: keaf301_Supplementary_Data [file keaf301_supplementary_data.zip › keaf301_Supplementary_Data/rhe-25-0437-File002.pdf]

Supplementary online material

Data S1. Treating Raynaud's disease with FSM

A) Rationale for treating Raynaud's disease with FSM

B) Using FSM to modify autonomic nervous system activity: case reports and presentations

Data S2. FSM treatment details

Data S3. Schedules and timings of administration of Cochin hand function score questionnaires

Data S4. Details and schedule/timings of the Raynaud's visual analogue scale questionnaire

Data S5. Statistical methods: additional details

Data S6. Results: additional details

A) First pilot study

B) Second pilot study

C) Pilot studies combined

D) Component questions of the Cochin hand function questionnaire

Data S7. Supplementary table S1: Microcurrent frequencies (in Hz): their meaning and use

Data S8. Supplementary table S2: Patient characteristics for the two studies

Data S9. Supplementary table S3: Change in individual components of the Cochin hand function questionnaire from pre-treatment to post-treatment in both pilot studies combined ordered by the degree of change

Data S10. Additional references

Data S1. Treating Raynaud's disease with FSM

A) Rationale for treating Raynaud's disease with FSM

A previous case study, involving 4 patients (unpublished results, see following paragraph, section 1B) looked at using FSM to modify autonomic nervous system activity. It has been suggested that overactivity of the sympathetic nervous system is the most common cause of Raynaud's syndrome[S1], and that heart rate variability (HRV) analysis can provide information on the balance between sympathetic and parasympathetic activity in the autonomic nervous system[S2]. When the body is exposed to cold temperatures the sympathetic nervous system releases norepinephrine and vasoconstricting neuropeptides to constrict the smooth muscle of the arterioles and reduce blood flow to the skin[S2]. In the aforementioned case report, the

frequency pair 40 Hz & 562 Hz when used together reduced the activity of the sympathetic nervous system as evaluated by HRV measurements. Given the pathology involved in Raynaud's syndrome it seemed appropriate to evaluate this frequency combination in this disease.

B) Using Frequency Specific Microcurrent (FSM) to modify autonomic nervous system activity: case reports and presentations. Roger Billica, February 2013 (with permission).

The autonomic nervous system (ANS) has autonomous control of key body functions, and consists of 3 parts:

A. Sympathetic = danger response, "flight or fight"

B. Parasympathetic = support for sleep, digestion, healing

C. Enteric = digestive system

The ANS can be linked with pH balance.

Patients with chronic disease will typically be Sympathetic "on" and Parasympathetic "off", with possible exceptions such as blood cancers.

Some of the causes of the sympathetic nervous system being "on" include a) actual danger; b) fear, stress, anxiety (what might be termed "feedback trap"); c) neurotoxins (e.g. biotoxins, aspartame, MSG, Mercury); d) trauma/emotions stored in fascia; e) acidic pH; f) lack of sunshine, lack of "grounding"; g) chronic pain; h) head trauma, loss of cranial-sacral pump; i) malnutrition, zinc deficiency; j) guilt; k) infections (acute or chronic); l) low-cholesterol (cannot make hormones).

Some of the consequences of the Sympathetics being "on" and the Parasympathetics being "off" include: a) over-production of stimulating neurotransmitters; b) digestion is turned off (resulting in poor enzyme production, malabsorption of nutrients, supplements etc); c) overgrowth of parasites and candida; d) adrenal stress (high cortisol, high insulin); e) anxiety, insomnia, ADD; f) hypertension, tachycardia

Is it possible to alter expression of the autonomic nervous system (and affect symptoms) through the use of Frequency Specific Microcurrent? Frequencies are known from the 1922 list (see main manuscript) to be 562 Hz for the sympathetic nervous system and 709 Hz for the parasympathetic nervous system. 40 Hz is thought to reduce activity, 49 Hz to increase vitality, and 81 Hz to increase secretions. We can use Heart Rate Variability measurements to evaluate the effects of FSM on the sympathetics and the parasympathetics. An example of a Heart Rate Variability report is seen in Supplementary Figure S1.

The first test case was a 58-year-old physician with chronic pain (neck), hypertension and a history of kidney stones. The protocol and evaluation were as follows:

- do baseline HRV, blood pressure and pulse (taken after lunch)

- run FSM: 40/709 for 1 minute; repeat measurements after a two-minute wait

- run FSM: 49, 81/562 for 1 minute each; repeat measurements after a two-minute wait
- run FSM: 49, 81/709 for 1 minute each; repeat measurements after a two-minute wait
- run FSM: 40/10 and 40/562 for 1 minute each; repeat measurements after a two-minute wait

The results can be seen in Supplementary Figure S2 and these results all seem consistent with the hypotheses raised earlier that 40/562 or 40/709 would reduce activity in that system, and that 49 & 81 would increase activity in that system.

Note that 40/10 is thought to reduce inflammation and activity in the spinal cord and is one of the recommended FSM approaches to dealing with chronic neck pain (*McMakin CR, Gregory WM, Phillips TM. Cytokine changes with microcurrent treatment of fibromyalgia associated with cervical spine trauma. Journal of Bodywork and Movement Therapies. 2005 (9) 169-176*).

The next case (Case #1), after the baseline case, was a 57-year-old female with an autoimmune hypothyroid condition; NO/ONOO syndrome with mitochondrial dysfunction (*intolerant of thyroid replacement); anxiety/stress; and hypertension.

The HRV results for this case can be seen in Supplementary Figure S3.

The baseline HRV for this patient shows that she was very sympathetic dominant. As can be seen, an FSM treatment consisting of 40/562, 49/709, 81/709 lowered sympathetic activity and elevated parasympathetic activity - the patient felt "tired, sleepy". Then FSM settings of 49/562, 81/562 (which was brief) normalised the sympathetic/parasympathetic balance. Subsequently, and long-term, the patient used 49,81/709 for calming and sleep support.

The next case (Case #2) was a 52 year old female with refractory insomnia, which had failed all treatments. She also had fatigue, osteopenia, and poor adrenal function. The initial HRV analysis showed that she was very nearly ALL sympathetic, almost NO parasympathetic. Her HRV results can be seen in Supplementary Figure S4.

The patient was given an initial FSM treatment of 40/562 and 49, 81/709. As a result, the patient fell asleep on the table and felt very relaxed (which was not typical for her). The next day before having FSM her HRV showed an even better balance, and the patient felt good. Repeat FSM treatments over the next several days maintained balance and stability of the autonomic nervous system. The long-term result was better sleep (though not perfect) and less anxiety.

The next case (Case #3) was a 50-year-old female having post-traumatic brain injuries, with PTSD. She also had Grave' s disease with hypothyroidism, adrenal hypofunction, multiple chemical sensitivities, and mold/fungus biotoxin illnesses.

Her HRV reports can be seen in Supplementary Figure S5.

Her baseline pre-FSM HRV chart shows a sympathetic dominant pattern. An FSM treatment consisting of 49, 81/709 (we only tried to activate the parasympathetics without turning off the sympathetics) resulted in a better balance in the autonomic nervous system. The patient said she felt more like her "old self".

The next case (Case #4) was a 70-year-old female with chronic lymphocytic leukaemia. She was stable on routine IV vitamin C infusions. She was also hypothyroid and had inflammatory arthritis.

Her HRV reports can be seen in Supplementary Figure S6.

For case #4 her baseline HRV shows that she was very parasympathetic dominant. She went on a metabolic typing diet and afterwards the balance was slightly better, but she was still parasympathetic dominant. After being given FSM settings of 49, 81/562 she was much more balanced, as seen in the last HRV figure, and said that she felt more alert and had more energy.

In conclusion, Frequency Specific Microcurrent appears to be effective in helping a patient to balance expression of the Autonomic Nervous System (as measured by Heart Rate Variability testing) and helps alleviate symptoms of ANS imbalance.

Data S2. FSM treatment details

For the treatment of the scleroderma, electrodes were attached to warm wet cloths positioned around the patient's neck and hands so that the microcurrent and frequencies passed through the arms and hands. The hands and forearms were massaged continuously by the treating practitioner to assist the frequencies in breaking down the scarring, as shown in supplementary figure S7. The frequency to "reduce scarring" is thought to resonate with and loosen the bonds holding the scar tissue in a shortened configuration. Massage was continued throughout treatment to disrupt the loosened bonds and release the scar tissue, generally for a few minutes at each frequency. No patients experienced negative side effects either during or following treatment.

Data S3. Schedules and timings of administration of Cochin hand function score questionnaires.

In both trials, the questionnaires were administered immediately prior to the first treatment. In the first trial, the post treatment questionnaire was administered on day 2, and one week, one month and three months after the first treatment. In the second trial, the questionnaire was filled out pre-treatment and immediately after treatment on day one and then after 1 week, 2 weeks and 3 weeks.

Data S4. Details and schedule/timings of the Raynaud's visual analogue scale questionnaire

The patients rated the severity of their Raynaud's disease using a simple visual analogue scale, both pre-treatment, and again at 1 week, 2 weeks and 3 weeks. Changes were evaluated in relation to the MCID and the patient acceptable symptom state (PASS) as evaluated by Khanna et al.[S3] who reported an MCID of 14 or 15 points and a PASS of 34 points. The follow-up forms were posted to the patients, with a stamped addressed envelope for return. A relatively small number of patients returned the follow up questionnaires for reasons that are not known.

Data S5. Statistical methods: additional details

All reported p-values are 2-sided. Data was missing on a number of patients, so, where appropriate, the linear increment method[S4] was used to extrapolate beyond the follow-up of an individual patient. This method involves predicting the pattern of change for the individual patient beyond their own follow-up by allowing each individual's value to follow the overall (linear) pattern of the whole cohort from one time point to another [S4]. So there is an imputed value for each missing data item which is the last value plus the mean change in the overall group from the time point at which that value was recorded to the next time point. Thus, the mean values are not compromised by dropouts. The standard error (SE) of the mean and, therefore, the conference interval (CI) on the mean are based on the number of actual values at that time point; values carried forward are used in calculating the mean, but not the SE. This method was only required with one analysis, the results of which are shown in figure 2C.

Data S6. Results: additional details

A. First pilot study

Improvements were largely maintained in 2 of the 3 patients who exhibited very substantial improvements up to 3 months, with one patient deteriorating back to pre-treatment levels. Overall, for the 6 patients treated, pre-treatment to post-treatment improvements did not reach statistical significance ($p = .06$, Wilcoxon test). Another patient with a lesser response and a higher initial value had severe arthritis in her hands and fingers in addition to the scleroderma, so would not be expected to respond to frequencies for scarring in the skin. The patient with the worst pre-treatment score was unable to attend for the second session, and her score at 1 week showed only marginal improvement from the pre-treatment score, so her post-treatment score was assumed to be the same as her pre-treatment score, since no patients reported worse scores after treatment. For all 5 patients who showed improvements the improvements were greater than the MCID, with 3 patients showing improvements of more than 5 times the MCID.

B. Second pilot study

The score of the patient with the worst score, rated extremely high/severe at 64 out of a maximum possible score of 90, remained unchanged. Six of the 11 patients had improvements greater than the MCID, 2 had improvements less than the MCID and 2 patients had starting scores less than the MCID making it impossible to achieve the MCID. Follow-up data for the Cochin hand function score over the 3 weeks following the treatment day was incomplete. Only 4 of the 11 patients returned their questionnaires.

The mean improvement when comparing pre-treatment values with those at one week is greater than the MCID, though only 2 patients had individual improvements greater than the MCID. However, these 2 patients had considerable improvements of 25 and 28 points. Four patients had starting scores above the PASS threshold of 34, and 2 of these showed improvements that reduced their scores to below this threshold, so, by this definition, their Raynaud's was reduced in severity to an acceptable state. Using the linear increment method[20] it was possible to plot the changes in Raynaud's VAS over the 3 weeks following treatment, allowing appropriately for the missing data (figure 2C). The confidence intervals get wider as there is more missing data, but the graph suggests that improvements are largely maintained, though there is a degree of worsening over time.

C. Pilot studies combined

Note that the two patients with the highest scores (64 & 57) showed little or no improvement, though there was no suggestion of a correlation between starting value and degree of improvement in the other patients.

D. Component questions of the Cochin hand function questionnaire

Responses to component questions of the Cochin hand function questionnaire, over both pilot studies combined, indicates which hand functions/issues the treatment changed most effectively (see supplementary table S3). The order of the questions in the table suggests that the treatment may be creating increased sensitivity and motor activity in the fingers/hands. Changes in these functions would explain the immediate improvement in ability to pick up coins from a tabletop, for which two patients reported improvements of 4 points on the form, from “Nearly impossible to do” before treatment to “Yes, without difficulty” after treatment, as detailed in the manuscript. There were also 9 instances of improvements of 3 points on the form, from “Nearly impossible to do” to “Yes, with a little difficulty” or from “Yes, with much difficulty” to “Yes, without difficulty”.

Data S7. **Supplementary table S1.** Microcurrent frequencies (in Hz): their meaning and use[12]

**Conditions:**	**Tissues:**	**Combination frequencies:**
13: scarring, 3: sclerosis	355: skin	13/355, 3/355
58: degeneration (occurs later)	162: capillaries	58, 13, 51/62, 77, 191, 480, 162
51: fibrosis	77: connective tissue	91, 217/77, 62, 142
40: inflammation	62, 142: muscle	58/00, 02, 32
124: something torn or broken	480: joint capsule<br>191: tendons	124/191, 77
91,217: calcium/mineral deposits	562: sympathetic nervous system	40/562 - Raynaud’s

Data S8. **Supplementary table S2**. Patient characteristics for the two studies.

	Variable	Result	Number of patients
Study 1	Age (mean, range)	61.5 (53-76)	6
	mRSS (mean, range)	3.5 (0-9)	4 (2 unknown)
	Capillaroscopic Pattern	Early	2
		Late	2
		Active	2
	Digital Ulcers	Absent	5
		Unknown	1
Study 2			
	Age (mean, range)	61.8 (42-78)	11
	mRSS (mean, range)	4.6 (0-15)	10 (1 unknown)
	Capillaroscopic Pattern	Absent	1
		Early	2
		Late	4
		Non-specific	2
		Unknown	2
	Digital Ulcers	Absent	7
		Present	3
		Unknown	1

Concomitant therapies used in the two studies:

Abatacept, Alendronate, Aspirin, Badesinide, Bosentan, BuTrans Patch, Clopidogrel, Co-codamol, Codeine, Calcium, Colesevelam, Depo-Medrone, Esomeprazole, Erythromycin, Famotidine, Fexofenadine, Fluoxetine, Furosemide, Gaviscon, Hydroxychloroquine, HYLO-Forte eyedrops, Iloprost infusions, Lactulose, Lansoprazole, Lisinopril, Loperamide, Losartan, Lyemecycline/Minocycline, Mebeverine, Metoclopramide, Movicol, Multivitamins, Mycophenolate, Nifedapine/Nifedipress, Nintedanib, Omeprazole, Ondansetron, Paracetamol, Pilocarpine, Prednisone, Pregabalin, Quetiapine, Ramipril, Ranitidine, Rosuvastatin, Ruminide, Refaximin, saline gel, Salivix, Senna, Sertraline, Sildenifil, Thyroxine, Tocilizumab, Tramadol, Ursodeoxycholic Acid, Venesection, Vitamin D

Data S9. **Supplementary table S3.** Change in individual components of the Cochin hand function questionnaire from pre-treatment to post-treatment in both pilot studies combined ordered by the degree of change

Cochin hand function question	mean change*	maximum change*
Can you pick up coins from a tabletop?	1.118	4
Can you turn a round door knob?	0.765	3
Can you squeeze a new tube of toothpaste	0.765	2
Can you unscrew the lid from a jar that has been opened before?	0.765	3
Can you turn a key in a lock?	0.706	3
Can you button your shirt?	0.706	2
Can you write a letter with an ordinary pen?	0.676	3
Can you peel fruit?	0.618	3
Can you cut a piece of paper with scissors?	0.529	2
Can you open and close a zipper?	0.529	2
Can you prick things well with a fork?	0.529	2
Can you write a short sentence with an ordinary pen?	0.500	2
Can you cut meat with a knife?	0.471	3
Can you grasp a full bottle and raise it?	0.412	2
Can you hold the toothbrush effectively?	0.353	2
Can you pour liquid from a bottle into a glass?	0.353	2
Can you hold a plateful of food?	0.353	2
Can you hold a bowl?	0.353	2

Data S10. Additional references

S1. Kubilay K, Kaya E, Kadan M, Arslan G, Demirkilic U, Celic M. Autonomic imbalance assessed by time-domain heart rate variability indices in primary Raynaud's phenomenon. Cardiovasc J of Afr 2015; 26(6): 214-216.

S2. Musa R, Qurie A. Raynaud Disease. [Updated 2023 Aug 8]. In: StatPearls [Internet]. Treasure Island (FL): StatPearls Publishing; 2024 Jan-. Available from: https://www.ncbi.nlm.nih.gov/books/NBK499833/

S3. Khanna PP, Maranian P, Gregory J, Khanna D. The minimally important difference and patient acceptable symptom state for the Raynaud's condition score in patients with Raynaud's phenomenon in a large randomised controlled clinical trial. Ann Rheum Dis. 2010 Mar;69(3):588-91.

S4. Diggle PJ, Farewell DM, Henderson R. Analysis of longitudinal data with drop-out: objectives, assumptions and a proposal. J R Stat Soc Ser C Appl Stat. 2007; 56:499-550
