# Supplementary material for: Frequency-specific microcurrent improves hand function and Raynaud’s symptoms in scleroderma: results of two pilot studies
Source: Rheumatology (Oxford). 2025 Jun 4;64(10):5504–8. doi: 10.1093/rheumatology/keaf301 (PMC12494225; doi:10.1093/rheumatology/keaf301)
Supplement: keaf301_Supplementary_Data [file keaf301_supplementary_data.zip › keaf301_Supplementary_Data/rhe-25-0437-File004.pdf]

Test Case: Baseline. 58 years old physician post-prandial. BP 140/90, Pulse 80:

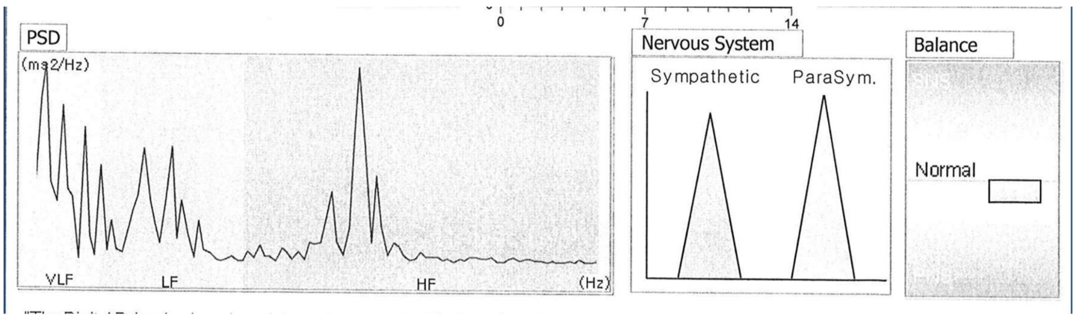

Test Case: FSM = 40/709 BP 140/90 P 80:

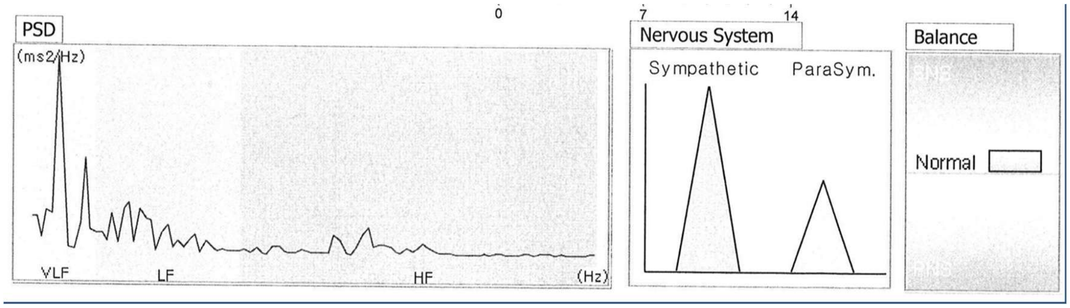

Test Case: FSM = 49,81/562 BP 150/90 P 89. Patient feels more awake, alert:

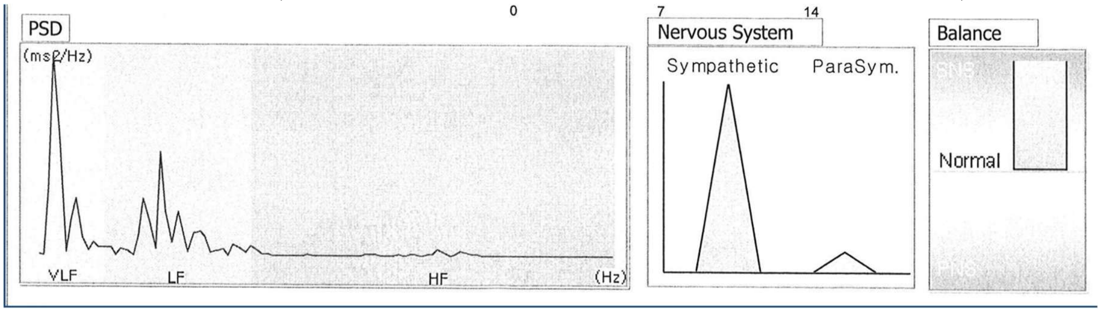

Test Case: FSM = 49,81/709 BP 144/90 P 80. Patient feels relaxed, sleepy:

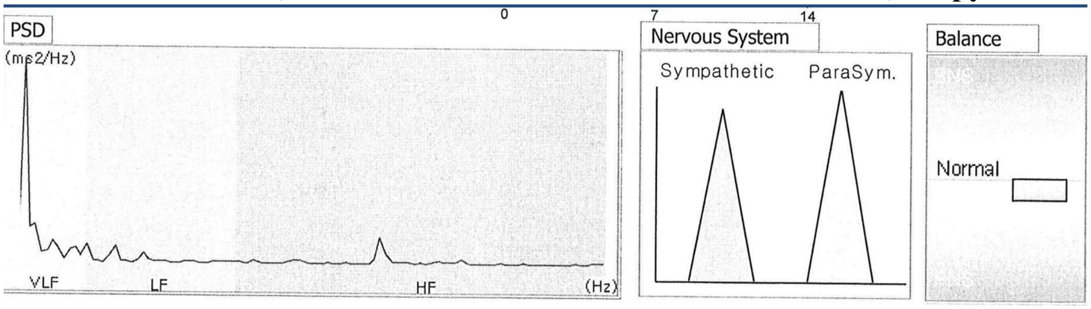

Test case: FSM = 40/10 and 40/562 BP 140/90 P 80 - end of study:

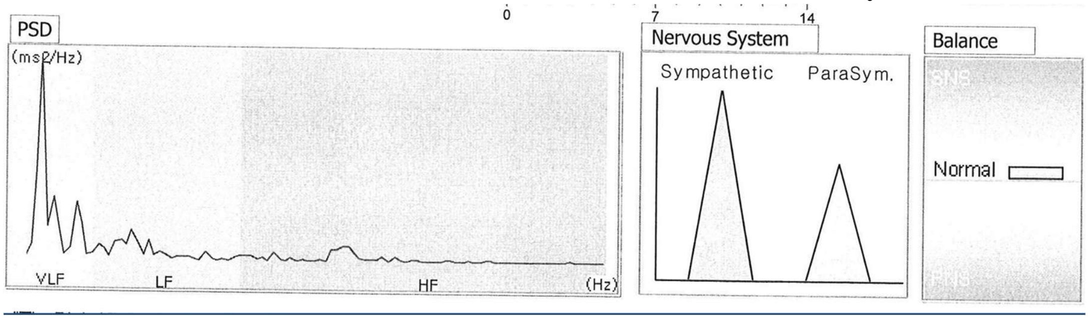

Supplementary Figure S2. Test case HRV reports
