# Supplementary material for: Frequency-specific microcurrent improves hand function and Raynaud’s symptoms in scleroderma: results of two pilot studies
Source: Rheumatology (Oxford). 2025 Jun 4;64(10):5504–8. doi: 10.1093/rheumatology/keaf301 (PMC12494225; doi:10.1093/rheumatology/keaf301)
Supplement: keaf301_Supplementary_Data [file keaf301_supplementary_data.zip › keaf301_Supplementary_Data/rhe-25-0437-File006.pdf]

Case #2: Baseline

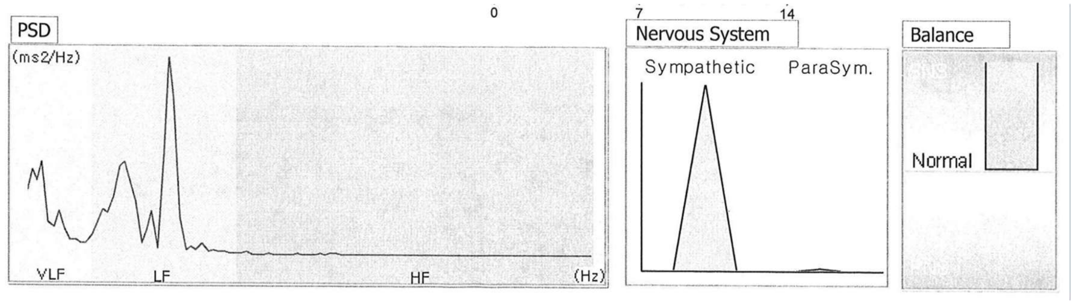

Case #2: FSM = 40/562 and 49,81/709

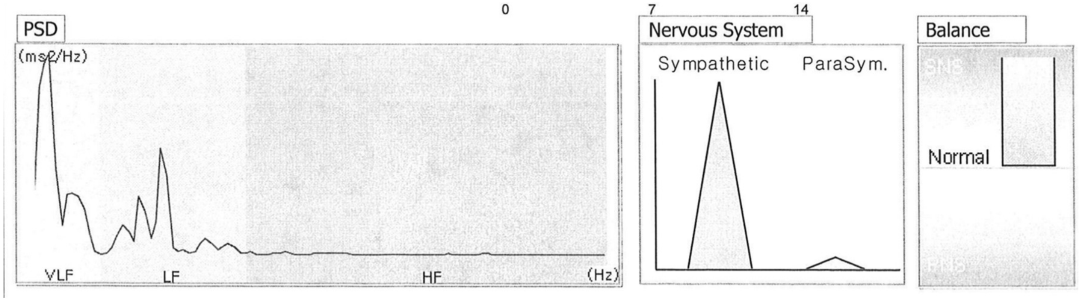

Case #2: Repeat FSM = 40/562 and 49,81/709

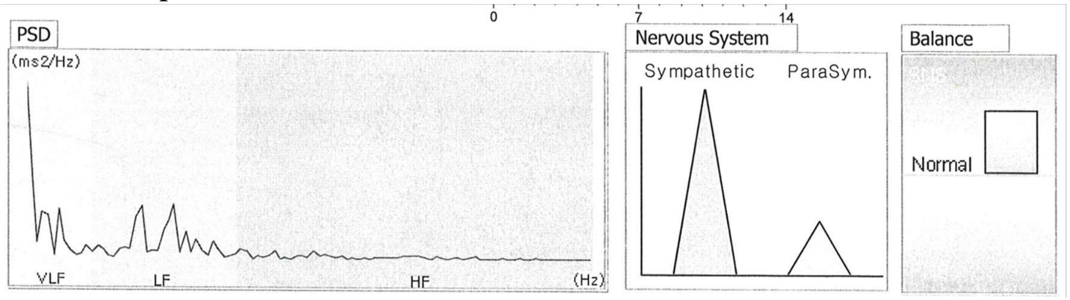

Case #2: Repeat FSM = 40/562 and 49,81/709

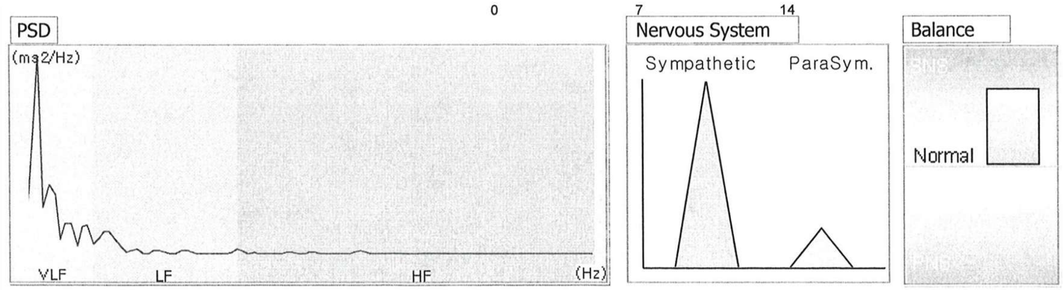

Supplementary Figure S4. Case #2 HRV reports
