# Supplementary material for: Frequency-specific microcurrent improves hand function and Raynaud’s symptoms in scleroderma: results of two pilot studies
Source: Rheumatology (Oxford). 2025 Jun 4;64(10):5504–8. doi: 10.1093/rheumatology/keaf301 (PMC12494225; doi:10.1093/rheumatology/keaf301)
Supplement: keaf301_Supplementary_Data [file keaf301_supplementary_data.zip › keaf301_Supplementary_Data/rhe-25-0437-File008.pdf]

Case #4: Baseline

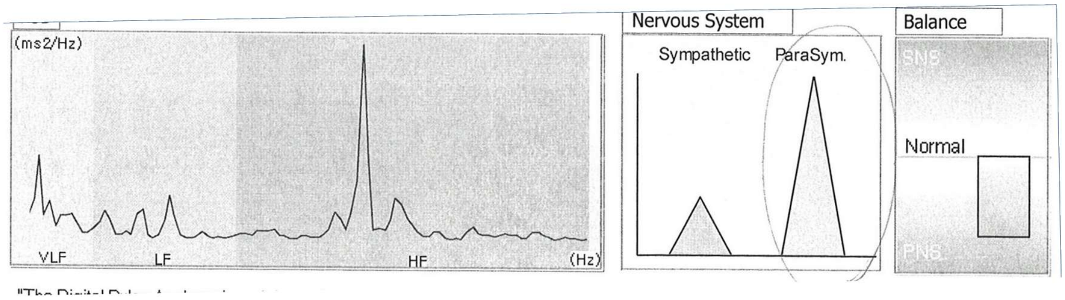

Case #4: After "metabolic typing" diet intervention

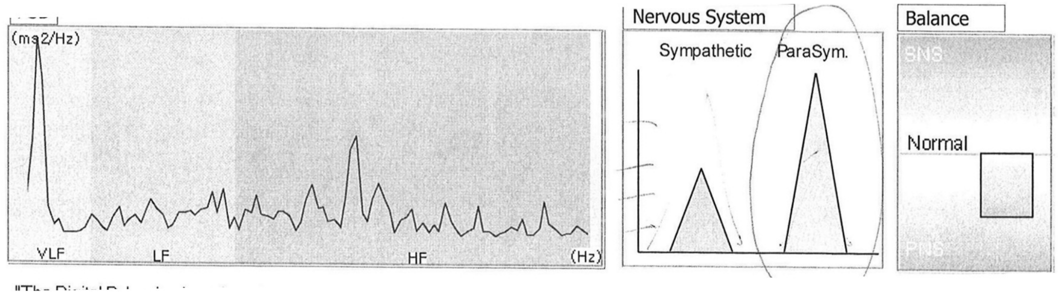

Case #4: FSM = 49,81/562. Patient feels more alert, more energy

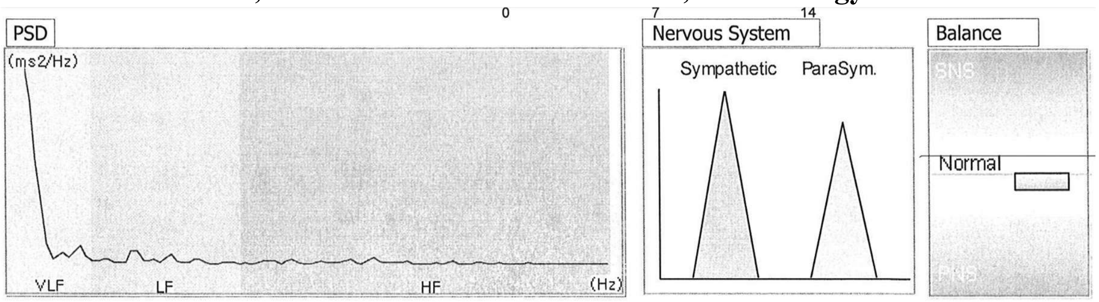

Supplementary Figure S6. Case #4 HRV reports
