# Supplementary material for: Frequency-specific microcurrent improves hand function and Raynaud’s symptoms in scleroderma: results of two pilot studies
Source: Rheumatology (Oxford). 2025 Jun 4;64(10):5504–8. doi: 10.1093/rheumatology/keaf301 (PMC12494225; doi:10.1093/rheumatology/keaf301)
Supplement: keaf301_Supplementary_Data [file keaf301_supplementary_data.zip › keaf301_Supplementary_Data/rhe-25-0437-File009.docx]

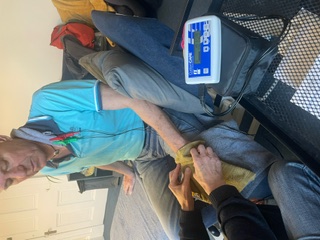


**Supplementary Figure S7**. A patient having his hands massaged using the medical device, with leads connected from the medical device to a cloth around his neck and another cloth on his hand.
