# Supplementary figures and images for: Frequency-specific microcurrent improves hand function and Raynaud’s symptoms in scleroderma: results of two pilot studies
Source: Rheumatology (Oxford). 2025 Jun 4;64(10):5504–8. doi: 10.1093/rheumatology/keaf301 (PMC12494225; doi:10.1093/rheumatology/keaf301)

### Analysis with Diagram

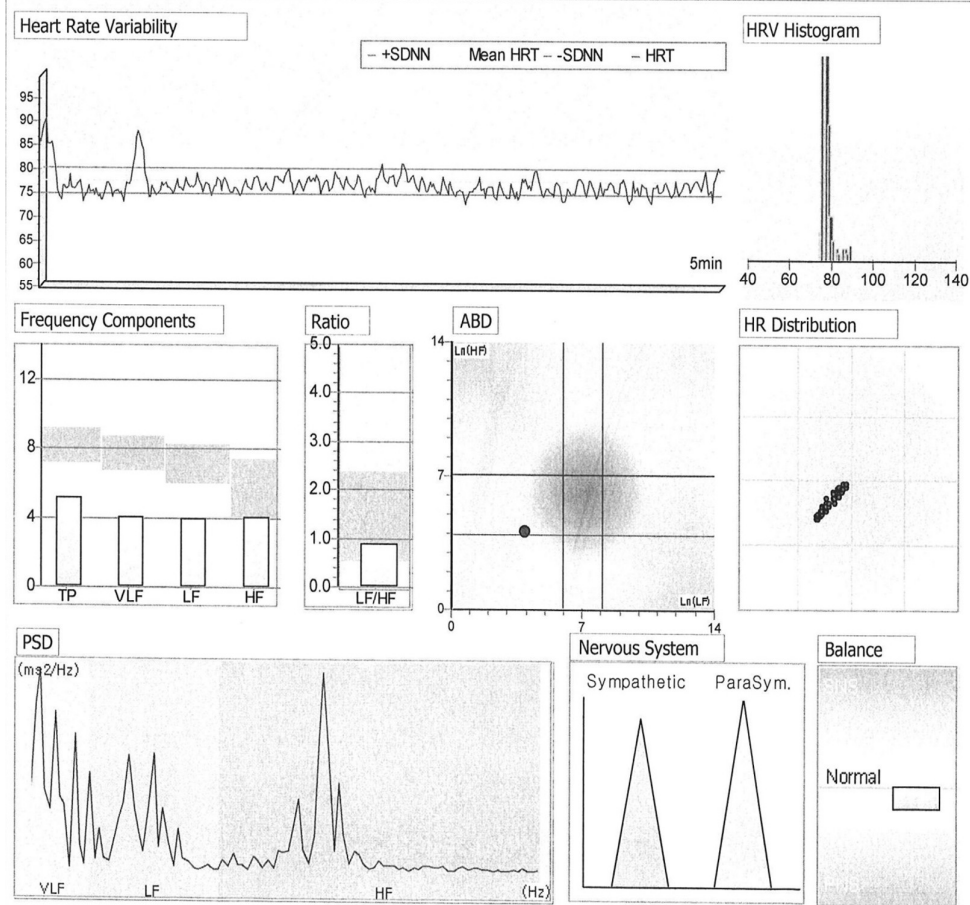

**Supplementary Figure S1. Example of Heart Rate Variability Test Report**

Supplement: keaf301_Supplementary_Data [file keaf301_supplementary_data.zip › keaf301_Supplementary_Data/rhe-25-0437-File003.pdf]

**Case #1: Baseline**

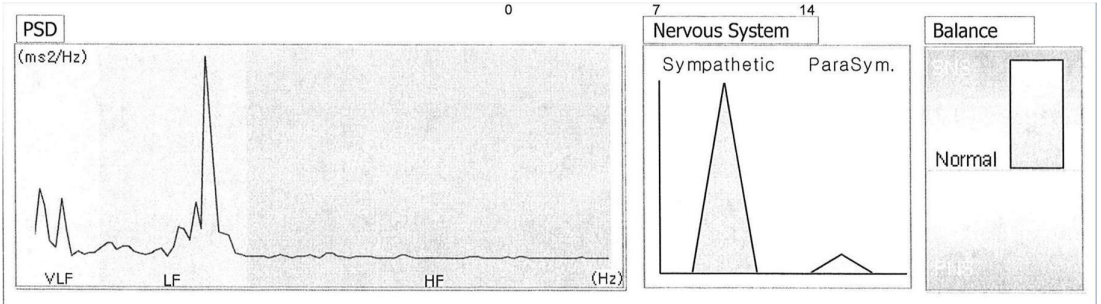

**Case #1: FSM = 40/562 and 49,81/709**

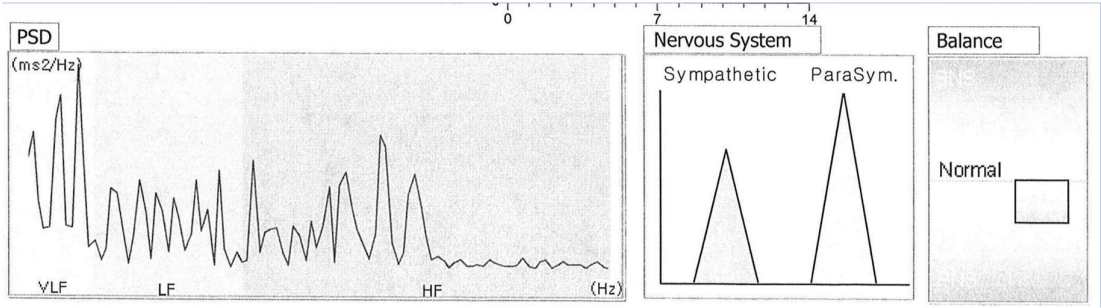

**Case #1: FSM = 49,81/562**

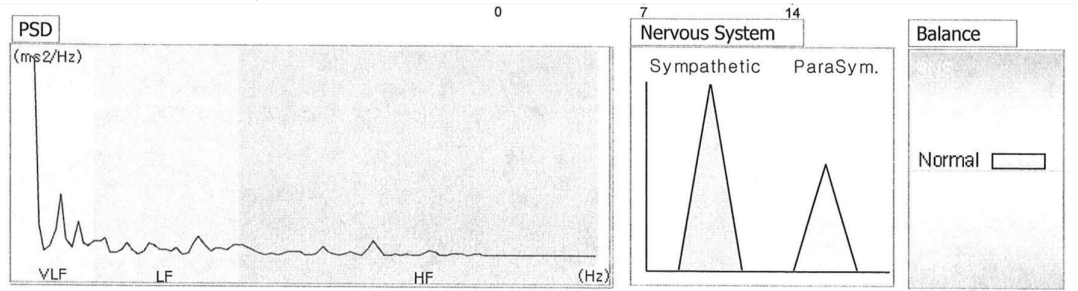

**Supplementary Figure S3. Case #1 HRV reports**

Supplement: keaf301_Supplementary_Data [file keaf301_supplementary_data.zip › keaf301_Supplementary_Data/rhe-25-0437-File005.pdf]

Case #3: Baseline

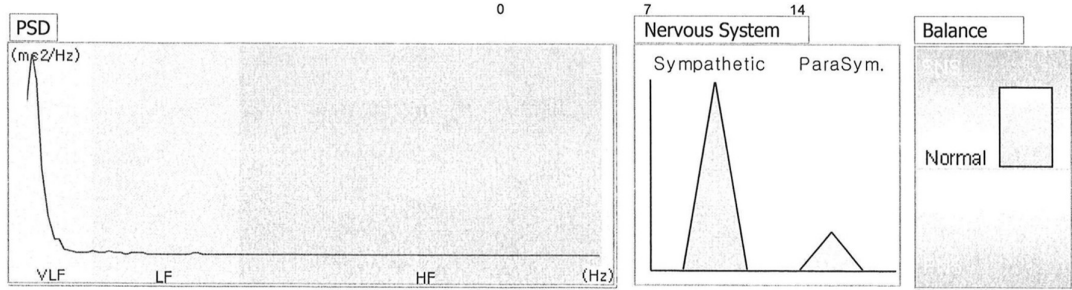

Case #3: FSM = 49,81/709

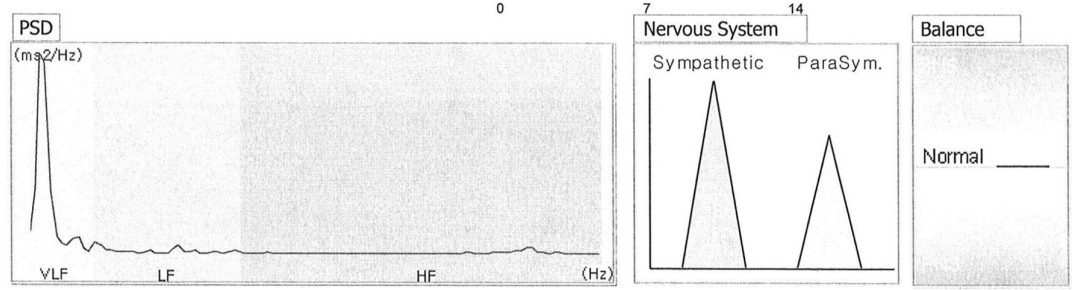

Supplementary Figure S5. Case #3 HRV reports

Supplement: keaf301_Supplementary_Data [file keaf301_supplementary_data.zip › keaf301_Supplementary_Data/rhe-25-0437-File007.pdf]
